# SBRT vs HDR Brachytherapy for Intermediate-Risk Prostate Cancer

**DOI:** 10.1001/jamanetworkopen.2026.0146

**Published:** 2026-02-25

**Authors:** Cristian Udovicich, Patrick Cheung, William Chu, Hans Chung, Jay Detsky, Stanley Liu, Gerard Morton, Chia-Lin Tseng, Danny Vesprini, John M. Hudson, Wee Loon Ong, Thomas Kennedy, Joelle Helou, Melanie Davidson, Ananth Ravi, Merrylee McGuffin, Liying Zhang, Alexandre Mamedov, Andrea Deabreu, Meghan Kulasingham-Poon, Andrew Loblaw

**Affiliations:** 1 Odette Cancer Centre, Sunnybrook Health Sciences Centre, Toronto, Ontario, Canada; 2 Department of Radiation Oncology, University of Toronto, Toronto, Ontario, Canada; 3 GenesisCare Radiation Oncology, Melbourne, Victoria, Australia; 4 Alfred Health Radiation Oncology, School of Translational Medicine, Monash University, Melbourne, Victoria, Australia; 5 BC Cancer, Kelowna, British Columbia, Canada; 6 Division of Radiation Oncology, Verspeeten Family Cancer Center, Western University, London, Ontario, Canada; 7 Computer Science, Mathematics, Physics and Statistics Department, University of British Columbia Okanagan, Kelowna, British Columbia, Canada; 8 Department of Health Policy, Measurement and Evaluation, University of Toronto, Toronto, Ontario, Canada

## Abstract

**Importance:**

Stereotactic body radiotherapy (SBRT) and high–dose-rate brachytherapy monotherapy (HDR-BT) are options for intermediate-risk prostate cancer. However, no prospective evidence is available to compare these modalities.

**Objective:**

To compare the biochemical failure (BCF), late patient-reported quality of life (PR-QoL), and acute and late adverse events (AEs) associated with SBRT and HDR-BT using prospective data.

**Data Sources:**

This was an individual patient data post hoc pooled analysis of 5 prospective trials with recruitment from 2010 to 2018. Statistical analyses were performed in September 2024.

**Study Selection:**

This was a post hoc analysis of these 5 sprospective trials. Eligibility criteria comprised men with intermediate-risk prostate cancer undergoing 5- or 2-fraction SBRT or 2-fraction HDR-BT. No androgen deprivation therapy was permitted.

**Data Extraction and Synthesis:**

Baseline patient and clinicopathological characteristics were requested.

**Main Outcomes and Measures:**

BCF, a minimal clinically important change on the PR-QoL, and clinician-reported AEs were the main outcomes.

**Results:**

After a median (IQR) follow-up of 9.5 (5.5-10.6) years, 247 men met the eligibility criteria, including 180 men undergoing SBRT (72.8%; mean [SD] age, 69.5 [6.7] years) and 67 men undergoing HDR-BT (27.1%; mean [SD] age, 66.0 [6.5] years). HDR-BT was associated with increased BCF. At 5 years, BCF was 7.8% (95% CI, 1.0%-14.6%) for HDR compared with 3.0% (95% CI, 0.4%-5.6%) for SBRT. At 10 years, BCF was 38.0% (95% CI, 19.8%-56.1%) for HDR compared with 10.4% (95% CI, 4.3%-16.6%) for SBRT (*P* < .001). The HDR-BT cohort had a significantly higher incidence of acute grade 2 or greater genitourinary AEs compared with SBRT (50 men [74.6%] vs 31 men [51.7%]; *P* = .007). There were no significant differences in any other acute or late AEs or late PR-QoL.

**Conclusions and Relevance:**

This post hoc pooled analysis reports a long-term comparison of SBRT and HDR-BT using prospective data. SBRT had significantly lower BCF and acute genitourinary AEs, and there was no significant difference in late PR-QoL.

## Key Points

**Question:**

Is stereotactic body radiotherapy (SBRT) or high–dose-rate brachytherapy monotherapy (HDR-BT) associated with better outcomes in men with intermediate-risk prostate cancer?

**Findings:**

In this post hoc pooled analysis of 5 prospective trials among 247 men, HDR-BT was associated with worse biochemical failure and a significantly higher incidence of acute grade 2 or higher genitourinary adverse events vs SBRT.

**Meaning:**

This is study’s long-term outcomes, including that SBRT was associated with decreased biochemical failure, may inform discussions regarding radiation modality selection for intermediate-risk prostate cancer.

## Teaser

This post hoc pooled analysis compares outcomes associated with stereotactic body radiotherapy (SBRT) vs high–dose-rate brachytherapy monotherapy (HDR-BT) among men with intermediate-risk prostate cancer.

## Introduction

Ultrahypofractionated radiotherapy with stereotactic body radiation therapy (SBRT) or high–dose-rate brachytherapy monotherapy (HDR-BT) are potential management options for intermediate-risk prostate cancer (IR-PCa) in National Comprehensive Cancer Network guidelines. The treatments have similarities, including ablative techniques with high doses per fraction. This may have a radiobiological benefit compared with conventional fractionation by taking advantage of the low alpha-beta ratio of prostate cancer. Both modalities use tight margins to minimize organ at risk dose. Additionally, they have reduced treatment time, which decreases the burden on patients and the health care system. Population-based studies of practice patterns have observed an increase in SBRT and a decrease in BT for IR-PCa.^1^

Two randomized clinical trials (RCTs)^2,3^ of men with mostly IR-PCa have demonstrated the noninferiority of SBRT or ultrahypofractionation compared with conventional or moderate hypofractionation. Compared with low dose-rate (LDR)–BT, single-fraction HDR-BT has demonstrated lower genitourinary (GU) adverse events (AEs) and higher patient-reported quality of life (PR-QoL) but worse prostate-specific antigen (PSA) response in a small phase 2 RCT.^4,5^ Two-fraction HDR-BT was found to have improved biochemical control compared with single-fraction HDR-BT.^6,7^

To our knowledge, no randomized evidence is available to compare HDR-BT and SBRT. The objective of this study was to perform a pooled analysis using individual patient data from 5 prospective trials to compare the incidence of biochemical failure (BCF), PR-QoL, and AEs associated with SBRT and HDR-BT for men with IR-PCa.

## Methods

### Study and Patient Selection

We performed an individual patient data post hoc pooled analysis from 5 prospective trials to compare Stereotactic Body Radiation Therapy vs High-Dose Rate Brachytherapy Monotherapy for Intermediate-Risk Prostate Cancer (SHERBET). These trials included men with low-risk or IR-PCa undergoing SBRT (4 trials, including 2 that were 5-fraction SBRT^8,9^ and 2 that were 2-fraction SBRT^10,11^) or HDT-BT (1 trial^7^). Three SBRT trials were nonrandomized,^8,9,10,11^ and the other trial was an RCT comparing weekly and every other day 5-fraction SBRT.^9^ The HDR-BT trial was an RCT comparing 2- vs single-fraction treatment.^7^ Detailed study designs and results of these trials were previously published.^6,8,9,10,11^ Trials were performed from 2010 to 2018. The formal trial follow-up was 5 years; however, patients were followed up as routine practice after this period. All contributing trials were approved by the Sunnybrook Research Ethics Board; 2 other research ethics boards (Cancer Care Manitoba and BC Cancer) approved 1 study.^9^ All participants provided written informed consent before enrollment.

This study was a post hoc analysis of these prospective trials. Eligibility criteria for this pooled analysis included patients with National Comprehensive Cancer Network (NCCN)–defined IR-PCa undergoing 5- or 2-fraction SBRT or 2-fraction HDR-BT. Patients receiving androgen deprivation therapy (ADT) in SBRT trials were excluded given that the HDR-BT trial did not allow ADT.

### Treatment Planning and Radiation Delivery

Detailed descriptions of treatment planning and radiation delivery have been published. In all SBRT trials,^8,9,10,11^ 3 gold fiducial markers were implanted transperineally into the prostate before computed tomography simulation. The dose prescription for 5-fraction trials^8,9^ was 40 Gy in 5 fractions to the clinical target volume (prostate only) with a 5-mm planning target volume (PTV) margin (PTV dose, D99 ≥38 Gy). The dose prescription for the 2-fraction trials^10,11^ was 26 Gy in 2 fractions to the clinical target volume (prostate with 1-cm seminal vesicle expansion) with a 2- to 3-mm PTV margin (PTV dose, D99 ≥22 Gy). One of the 2-fraction trials^11^ used a boost up to 32 Gy to the magnetic resonance imaging–defined dominant intraprostatic lesion (clinical target volume dose, D99 ≥ 26 Gy). Treatment was delivered using intensity-modulated radiation therapy in 5-fraction trials and volumetric-modulated arc therapy in 2-fraction trials. Daily image guidance with cone beam computed tomography was performed before and after treatment. An endorectal immobilization device (GU-Lok) was used in 2-fraction trials.^12^ Treatment was delivered every other day or weekly in 5-fraction trials and weekly in 2-fraction trials.

In the HDR trial,^6^ the dose prescription was 2 fractions of 13.5 Gy delivered 1 to 2 weeks apart. Treatment was delivered at a high-volume brachytherapy institution. The patient received treatment general anesthesia as an outpatient using intraoperative transrectal ultrasonography–based planning. The clinical target volume was the prostate with a 0- to 2-mm expansion. There was no further expansion to the PTV. Dwell time optimization was performed using Oncentra Prostate version 4.1.6 SP1 (Elekta). Planning objectives included the volume of PTV that gets 100% of the prescription (V100) greater than 95%, V150 less than 35%, or V200less than 12%; maximum dose (Dmax) to the urethra less than 120% or D10 less than 115%; and rectal Dmax less than 90% or V80 less than 0.2 cc. Dosimetric data have been published; the median PTV V100 was 97%, and the median V150 was 35%.^13^

### Data Extraction and End Points

Requested data consisted of baseline patient and clinicopathological characteristics. The primary end point was BCF. In all 5 trials,^6,8,9,10,11^ BCF was defined per the Phoenix definition (PSA nadir + 2.0 ng/mL). Secondary end points included late PR-QoL and acute or late AEs. Further secondary end points included PSA nadir, 4-year PSA response rate,^14^ distant metastases, cancer-specific survival (CSS), and overall survival (OS). PR-QoL was recorded using the complete Expanded Prostate Index Composite (EPIC). A minimal clinically important change was defined as an EPIC PR-QOL decrease greater than 0.5 SD. The decrease was calculated as the difference between baseline and mean EPIC score. AEs (GU, gastrointestinal [GI], and sexual AEs) were clinician reported using the Common Terminology Criteria for Adverse Events (CTCAE). One SBRT trial^9^ did not collect clinician-reported AEs using CTCAE criteria and could not be assessed. The HDR trial^6^ did not collect QoL data in the acute period (≤3 months); therefore, we could compare the 2 treatment modalities for PR- QoL only in the late period. Each trial period was 5 years. After this period, long-term BCF data were recorded at the time of visit or retrospectively. No formal assessment of AEs or PR-QoL was performed after the trial period.

### Statistical Analysis

Demographics and clinical characteristics were summarized in total patients and by 2 cohorts (SBRT vs HDR). The Wilcoxon rank-sum test, Fisher exact test, or χ^2^ test was applied to compare demographics and clinical characteristics, as appropriate. Median follow-up was calculated using the reverse Kaplan-Meier method.

Time-to-event end points were calculated in months from the date of baseline. For BCF, patients were censored at the time of event or last PSA recorded. For all other time-to-event outcomes (such as OS, distant metastases, and CSS), patients were censored at the time of event or last follow-up. For distant metastases, death was a competing event. For CSS, death from other causes was a competing event. Kaplan-Meier OS curves were created and compared by log-rank test. Cumulative incidence curves of BCF were estimated using the Nelson-Aalen method and compared by log-rank test. Cumulative incidence curves of distant metastases and CSS were estimated using the Fine-Gray method and compared by the Gray test. For BCF, exploratory comparisons were also conducted for patients from 5- vs 2-fraction SBRT vs HDR and favorable vs unfavorable intermediate risk. Univariate and multivariable Cox proportional hazard regression analyses were conducted for BCF. For the multivariable model, we included the following prespecified covariates: treatment (HDR-BT vs SBRT), PSA (≥10 vs <10 ng/mL), T stage (T1a-c vs T2a-c), and International Society of Urological Pathology grade group (3 + 3 vs 3 + 4, 3 + 3 vs 4 + 3, and 3 + 4 vs 4 + 3).

PR-QoL was summarized for domain scales by plotting mean and SD over months in patients from 2 cohorts. Proportions of patients with a minimal clinically important change were calculated for each domain and compared between 2 cohorts using the χ^2^ test or Fisher exact test, as appropriate. AEs were summarized and compared by the χ^2^ test or Fisher exact test, as appropriate. Cumulative incidence of late grade 2 or higher AEs was compared by the Gray test. Analyses of AEs and PR-QoL were conducted using a complete case approach. Missing data resulted from study-level differences in data collection (1 SBRT trial^9^ did not collect CTCAE data, and the HDR-BT trial^7^ did not collect acute PR-QoL) and were therefore considered structurally missing. Patients with available data for each end point were included in the corresponding analysis.

Statistical analyses were performed in September 2024. All analyses were conducted using SAS statistical software version 9.4 (SAS Institute) and R statistical software version 4.3.0 (R Project for Statistical Computing) packages. All statistical tests were 2-sided, and a *P* value < .05 was considered statistically significant.

## Results

There were 412 men in the 5 prospective trials,^6,8,9,10,11^ and 247 men met the eligibility criteria for this study. Of these, 180 men (72.8%) underwent SBRT (mean [SD] age, 69.5 [6.7] years) and 67 men (27.1%) underwent HDR-BT (mean [SD] age, 66.0 [6.5] years).

Regarding baseline characteristics, there were statistically and clinically significant differences for clinical T stage and NCCN risk group (Table 1). The SBRT cohort had a significantly higher proportion of men with higher T stage than the HDR-BT cohort (≥T2: 63 men [33.8%] vs 15 men [22.4%]; *P* = .03). The SBRT cohort had a significantly higher proportion of unfavorable IR-PCa compared with the HDR-BT cohort (112 men [62.2%] vs 26 men [38.8%]; *P* = .001). There was no significant difference in the median International Prostate Symptom Score at baseline.

**Table 1.** Demographic and Clinical Characteristics at Baseline

Characteristic	Men, No. (%) (N = 247)		*P* value
	HDR-BT (n = 67)	SBRT (n = 180)	
Age, mean (range), y	66.0 (49-80)	69.5 (53-85)	<.001
Clinical stage			
T1a-c	52 (77.6)	117 (65.0)	.03
T2a	15 (22.4)	42 (23.3)	
T2b	0	17 (9.4)	
T2c	0	2 (1.1)	
NA	0	2 (1.1)	
PSA at baseline, ng/mL			
Median (IQR)	6.4 (4.8-8.8)	7.9 (5.4-11.3)	.02
<10	55 (82.1)	126 (70.0)	.06
≥10	12 (17.9)	54 (30.0)	
ISUP grade group			
1: GS 3 + 3	3 (4.5)	11 (6.1)	.26
2: GS 3 + 4	57 (85.1)	135 (75.0)	
3: GS 4 + 3	7 (10.5)	34 (18.9)	
Positive cores, %			
<50	54 (80.6)	121 (67.2)	.04
≥50	13 (19.4)	59 (32.8)	
NCCN risk group			
Favorable intermediate risk	41 (61.2)	68 (37.8)	.001
Unfavorable intermediate risk	26 (38.8)	112 (62.2)	
Prostate volume at baseline, median (IQR), cc	35 (27-43)	36 (28-48)	.36
IPSS at baseline, median (IQR)	4 (2-8)	5 (3-10)	.28

Abbreviations: GS, Gleason score; HDR-BT, high–dose-rate brachytherapy; IPSS, International Prostate Symptom Score; ISUP, International Society of Urological Pathology; NCCN, National Comprehensive Cancer Network; PSA, prostate-specific antigen; SBRT, stereotactic body radiotherapy.

### BCF

**Figure 1.**
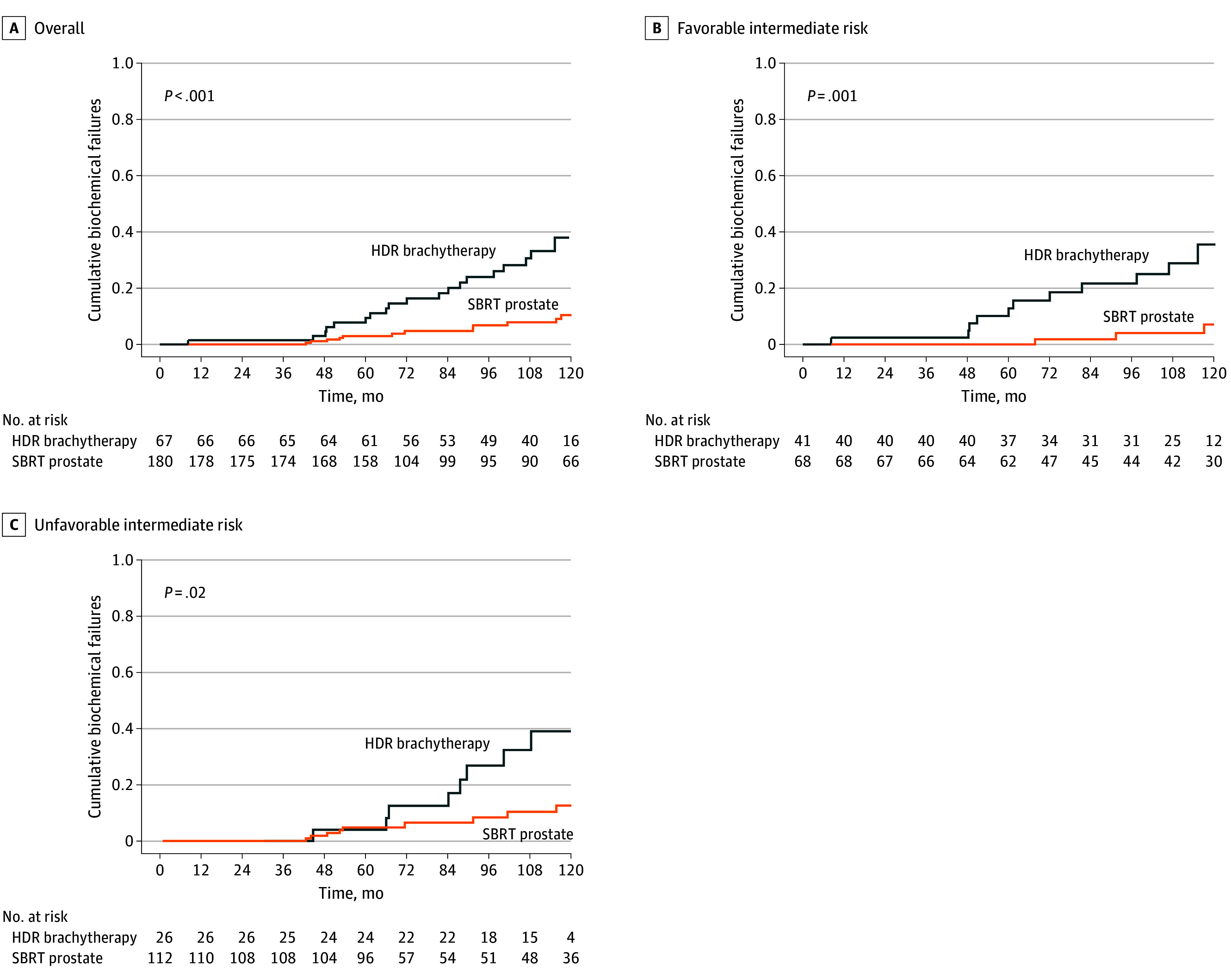
Cumulative Incidence of Biochemical Failure HDR indicates high dose rate; SBRT, stereotactic body radiotherapy.

The median (IQR) follow-up was 9.5 (5.5-10.6) years (Table 2). The cumulative incidence of BCF was significantly higher in the HDR-BT cohort. At 5 years, BCF was 7.8% (95% CI, 1.0%-14.6%) for HDR-BT compared with 3.0% (95% CI, 0.4%-5.6%) for SBRT. At 10 years, BCF was 38.0% (95% CI, 19.8%-56.1%) for HDR-BT compared with 10.4% (95% CI, 4.3%-16.6%) for SBRT (*P* < .001) (Figure 1A). For absolute BCF event counts, 19 patients (28.4%) in the HDR-BT cohort had a BCF compared with 13 patients (7.2%) in the SBRT cohort.

**Table 2.** Primary and Secondary Clinical Outcomes Among Patients Receiving HDR-BT vs SBRT

Outcome	Men, % (95% CI)		*P* value
	HDR-BT (n = 67)	SBRT (n = 180)	
Follow-up, median (IQR), mo	114.4 (108.8-121.1)	113.8 (64.0-132.1)	.42
PSA nadir, median (IQR), ng/mL	0.10 (0.03-0.42)	0.07 (0.02-0.19)	.14
Time to PSA nadir, median (IQR), mo	60.8 (35.9-100.3)	60.7 (45.2-99.1)	.53
4-y PSA response rate <0.4, No. %			
No	27 (40.3)	42 (23.3)	.02
Yes	38 (56.7)	123 (68.3)	
NA	2 (3.0)	15 (8.3)	
Cumulative incidence of biochemical failure			
At 5 y	7.8 (1.0-14.6)	3.0 (0.4-5.6)	<.001
At 8 y	24.0 (11.4-36.6)	6.8 (2.2-11.4)	
At 10 y	38.0 (19.8-56.1)	10.4 (4.3-16.6)	
Cumulative incidence of distant metastases			
At 5 y	4.6 (0-9.9)	0	.06
At 8 y	4.6 (0-9.9)	1.0 (0-2.9)	
At 10 y	9.4 (1.0-17.8)	3.1 (0-6.6)	
CSS			
At 5 y	100 (100-100)	99.4 (98.3-100)	.32
At 8 y	100 (100-100)	98.4 (96.3-100)	
At 10 y	100 (100-100)	98.4 (96.3-100)	
OS			
At 5 y	97.0 (93.0-100)	96.0 (93.2-99.0)	.13
At 8 y	94.0 (88.4-99.9)	87.5 (81.9-93.4)	
At 10 y	91.0 (84.3-98.1)	83.0 (76.7-89.9)	

Abbreviations: CSS, cancer-specific survival; HDR-BT, high–dose-rate brachytherapy; NA, not applicable; OS, overall survival; PSA, prostate-specific antigen; SBRT, stereotactic body radiotherapy.

BCF was further assessed in an exploratory analysis by IR-PCa risk group. HDR-BT was associated with increased BCF for favorable IR-PCa and unfavorable IR-PCa (Figure 1B and C)

The SBRT cohort was separated into 2- and 5-fraction SBRT for exploratory analysis (eFigure 1 in Supplement 1). There was no significant difference in BCF between 2- and 5-fraction SBRT (eTable 1 in Supplement 1). Individually, HDR-BT was associated with increased BCF compared with 2-fraction and 5-fraction SBRT.

On univariate analysis, factors associated with BCF included HDR-BT (hazard ratio [HR], 3.69; 95% CI, 1.80-7.55; *P* < .001), a PSA of 10 ng/mL or greater (HR, 2.33; 95% CI, 1.16-4.68; *P* = .02) and less than 50% of cores positive (HR, 0.27; 95% CI, 0.08-0.88; *P* = .03) (eTable 2 in Supplement 1). After multivariable analysis (eTable 3 in Supplement 1), significant covariates included HDR-BT (HR vs SBRT, 5.26; 95% CI, 2.46-11.25; *P* < .001) and a PSA of 10 ng/mL or greater (HR vs PSA <10 ng/mL, 3.86; 95% CI, 1.81-8.22; *P* < .001).

There was no significant difference between HDR-BT and SBRT in the median (IQR) PSA nadir (0.10 [0.03-0.42] ng/mL vs 0.07 [0.02-0.19] ng/mL; *P* = .14). There was a significantly lower proportion of men in the HDR-BT vs SBRT cohort with a 4-year PSA response rate (38 men [56.7%] vs 123 men [68.3%]; *P* = .02). There was no significant difference in the cumulative incidence of distant metastases, CSS, or OS (eFigure 2 in Supplement 1).

### Patient-Reported Quality of Life

The change from baseline EPIC score over time for each domain is depicted in eFigure 3 in Supplement 1. Comparative data were not available for the acute period. For the late period, there was no significant difference in PR-QoL. There was no significant difference in the proportion of patients in HDR-BT vs SBRT cohorts with a minimal clinically important change across the urinary domain, bowel domain, sexual domain, or hormonal domain (Table 3).

**Table 3.** Patient-Reported Quality of Life and AEs

Outcome	Men, No. (%) (N = 247)		*P* value
	HDR-BT (n = 67)	SBRT (n = 180)	
**MCIC on EPIC score**			
Urinary			
No MCIC	39 (59.1)	101 (60.1)	.89
MCIC	27 (40.9)	67 (39.9)	
Bowel			
No MCIC	46 (69.7)	108 (63.2)	.34
MCIC	20 (30.3)	63 (36.8)	
Sexual			
No MCIC	27 (42.9)	84 (56.0)	.08
MCIC	36 (57.1)	66 (44.0)	
Hormonal			
No MCIC	43 (67.2)	112 (71.8)	.50
MCIC	21 (32.8)	44 (28.2)	
**Clinician-reported CTCAE AEs**			
Acute GI grade ≥2			
No	64 (95.5)	59 (98.3)	.62
Yes	3 (4.5)	1 (1.7)	
Acute GU grade ≥2			
No	17 (25.4)	29 (48.3)	.007
Yes	50 (74.6)	31 (51.7)	
Acute sexual grade ≥2			
No	59 (88.1)	53 (88.3)	.96
Yes	8 (11.9)	7 (11.7)	
Late GI grade ≥2			
No	61 (91.0)	49 (81.7)	.12
Yes	6 (9.0)	11 (18.3)	
Late GU grade ≥2			
No	35 (52.2)	27 (45.0)	.42
Yes	32 (47.8)	33 (55.0)	
Late sexual grade ≥2			
No	33 (49.3)	29 (48.3)	.92
Yes	34 (50.8)	31 (51.7)	

Abbreviations: AE, adverse event; CTCAE, Common Terminology Criteria for Adverse Events; EPIC, Expanded Prostate Index Composite; GI, gastrointestinal; GU, genitourinary; HDR-BT, high–dose-rate brachytherapy; MCIC, minimal clinically important change; SBRT, stereotactic body radiotherapy.

### AEs

Data on CTCAE AEs were available for all men in the HDR-BT cohort and 60 men (33.3%) in the SBRT cohort (eTable 4 in Supplement 1). In the acute period, no grade 3 GI or GU AEs occurred. One grade 3 sexual AE occurred in the SBRT cohort. For acute grade 2 or higher GU AEs, the HDR-BT cohort had a significantly higher incidence compared with the SBRT cohort (50 patients [74.6%] vs 31 patients [51.7%]; *P* = .007) (Table 3). There was no significant difference in acute grade 2 or higher GI or sexual AEs.

**Figure 2.**
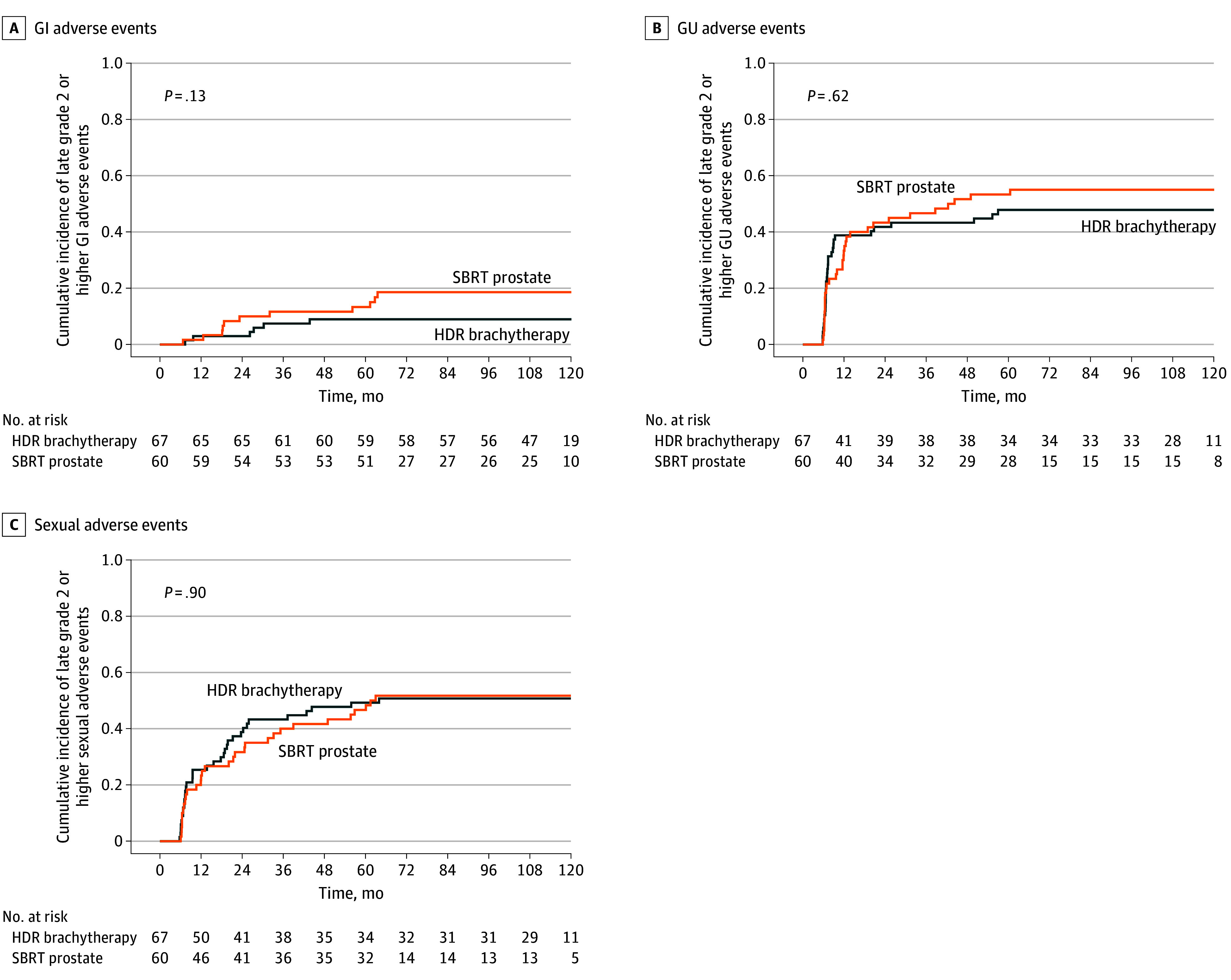
Cumulative Incidence of Grade 2 or Higher Late Adverse Events Grades were reported using Common Terminology Criteria for Adverse Events criteria. GI indicates gastrointestinal; GU, genitourinary; HDR, high dose rate; SBRT, stereotactic body radiotherapy.

In the late period, there were no grade 3 GI AEs. One grade 3 GU AE was recorded in the SBRT cohort. Grade 3 sexual AEs occurred in 2 men (3.3%) and 3 men (4.5%) men in SBRT and HDR-BT cohorts, respectively. In the late period, there was no significant difference between HDR-BT and SBRT groups in the cumulative incidence of late grade 2 or higher GI, GU, or sexual AEs (Table 3 and Figure 2).

## Discussion

In the SHERBET individual patient data post hoc pooled analysis of 5 prospective trials, we found a significantly increased risk of BCF and acute grade 2 or higher GU AEs with HDR-BT compared with SBRT. To our knowledge, this is the first comparison of these 2 modalities using prospective data. Other strengths of this study include individual patient data, consistent treatment protocols, a multivariable analysis, and an extended median follow-up.

Comparative evidence of HDR-BT and SBRT is limited to primarily retrospective data. A large retrospective multi-institutional study by Levin-Epstein et al^15^ compared SBRT (1716 patients), HDR-BT (512 patients), and LDR-BT (1274 patients) in men with low-risk prostate cancer and IR-PCa. No significant difference in BCF overall or within risk groups was observed. On univariate analysis, there was no significant difference in BCF for HDR-BT compared with SBRT (HR for HDR-BT, 1.74; 95% CI, 0.66-4.62; *P* = .27). A retrospective multi-institutional study by Hegde et al^16^ compared SBRT (300 patients) and HDR-BT (137 patients), with potential crossover of the patient population from Levin-Epstein et al.^15^ There was no significant difference in 5-year biochemical relapse–free survival for SBRT vs HDR-BT (95% vs 99%; *P* = .17). A single-institution study by Tsang et al^17^ reported on 185 men with mostly IR-PCa (96%). Data were available from a prospective trial for 2-fraction HDR-BT, and retrospective data were available for SBRT. No significant difference was observed in 5-year biochemical relapse–free survival between 2-fraction HDR-BT and SBRT (95% vs 92%; *P* = .37). However, limitations to these 3 studies include their retrospective nature (apart from 1 cohort in 1 study^17^), a shorter median follow-up (49-72 months), inconsistent use of ADT (not reported in Levin-Epstein et al,^15^ 0% in Hegde et al,^16^ and 19%-72% in Tsang et al^17^), different HDR-BT regimens (only 1 study^17^ had a 2-fraction HDR-BT cohort; 81%^15^ and 96%^16^ in the 2 other studies with potential crossover were 6-fraction HDR-BT cohorts), and that only 1 study^17^ reported outcomes in favorable and unfavorable IR-PCa.

Our study highlights that there may be a need for ongoing follow-up past 5 years for men with IR-PCa, particularly those undergoing HDR-BT. We are unable to provide a definitive answer for the difference in BCF, particularly given that SBRT and HDR-BT cohorts had a similarly high biologically effective dose. The HDR-BT dosimetric data were previously published and were in line with protocol requirements. Importantly, our prospective evidence mirrors other prospective evidence for each modality. The modest increase in BCF in the SBRT cohort in our study (3.0% at 5 years to 10.4% at 10 years) is similar to results of an analysis by Kishan et al^18^ of 12 prospective SBRT trials (7% at 5 years to 15% at 10 years). For HDR-BT, the considerable increase in BCF (7.8% at 5 years to 38.0% at 10 years) is similar to that demonstrated in the Mount Vernon prospective single-, 2-, and 3-fraction HDR-BT trial (4-year biochemical relapse–free survival, 91%-93% to 64%-76% at 10 years).^19,20^ However, it has to be noted that approximately 50% of men in the Mount Vernon trial had high-risk disease, and 73% to 87% received ADT.

Limited evidence is available to compare SBRT and HDR-BT for AEs and PR-QoL. We found a significant increase in acute grade 2 or higher GU AEs with HDR-BT but no significant difference in other acute or late AEs. In the previously mentioned combined retrospective and prospective study, Tsang et al^17^ reported statistically significantly increased late CTCAE grade 2 or higher GI AEs with SBRT (4% vs 2% for 2-fraction HDR-BT; *P* < .05). However, this 2% absolute difference is unlikely clinically meaningful. Of note, SBRT AEs were recorded retrospectively and the HDR-BT trial likely used Radiation Therapy Oncology Group criteria,^19^ which have been demonstrated to report lower grading compared with CTCAE.^3^ For PR-QoL, a retrospective study^21^ observed similar results to our pooled analysis, with no difference between SBRT and HDR-BT in any EPIC domain.

To our knowledge, there are no currently accruing RCTs comparing HDR-BT and SBRT. Men with unfavorable IR-PCa are eligible for the Androgen Suppression Combined With Nodal Irradiation and Dose Escalated Prostate Treatment (ASCENDE-SBRT) RCT evaluating HDR-BT boost and SBRT. Data from RCTs comparing LDR-BT and SBRT (A Trial Evaluating Toxicity of SBRT and LDRB in Localized Prostate Cancer) or LDR-BT and HDR-BT (A Randomized Phase II Trial of High Dose-Rate and Low Dose-Rate Brachytherapy as Monotherapy in Localized Prostate Cancer: Analysis of Initial Arms of Canadian Cancer Trials Group PR19 and Phase II High Dose Brachytherapy and Low Dose Rate Brachytherapy as Monotherapy in Localized Prostate Cancer) will also help inform the question of the optimal nonsurgical management of IR-PCa. PACE-A (SBRT vs radical prostatectomy) has reported PR-QoL and AE outcomes and will report oncologic outcomes in the next few years.^22^

### Limitations

Limitations of this pooled analysis include that this was a post hoc study of prospective trials, and caution should be applied to cross-trial comparisons. However, individual patient data enabled us to provide a robust comparison with multivariable modeling instead of an aggregate pooled analysis. There was heterogeneity regarding dose fractionation in the SBRT cohort (two 5-fraction, one 2-fraction without a dominant intraprostatic lesion boost, and one 2-fraction with a dominant intraprostatic lesion boost). However, we observed no significant difference in BCF between 5- and 2-fraction SBRT in this study or in previous analyses.^23^ Five- and 2-fraction SBRT were both associated with improved BCF compared individually with HDR-BT. No increase in biochemical control has been observed with a dominant intraprostatic lesion boost in 2-fraction SBRT.^10^ Caution should also be applied to BCF comparisons between intermediate-risk groups and 5- vs 2-fraction vs HDR-BT given that these were exploratory analyses. Additionally, we cannot make any firm conclusions about other HDR-BT regimens greater than 2 fractions. Interpretation of comparative AE findings should be made with caution given that the imbalance in CTCAE data availability (available for only approximately one-third of the SBRT cohort given that the largest trial used Radiation Therapy Oncology Group criteria) and the absence of acute PR-QoL data (not collected in the HDR-BT trial^7^) may have influenced observed differences in reported AEs. Although multivariable analysis was performed, residual confounding cannot be excluded given the nonrandomized design and baseline differences in disease characteristics.

## Conclusions

This SHERBET post hoc pooled analysis reports the first comparison of SBRT and HDR-BT using individual patient data from prospective trials. HDR-BT had significantly increased BCF and acute CTCAE grade 2 or higher GU AEs compared with SBRT. Based on our findings, these prospective data further support the role of SBRT for men with IR-PCa.

## Notes

Supplement 1. eTable 1. Cumulative Incidence of Biochemical Failure (HDR-BT vs 2-Fraction SBRT vs 5-Fraction SBRT) eTable 2. Univariate Analysis for Biochemical Failure eTable 3. Multivariable Analysis for Biochemical Failure eTable 4. Demographic and Clinical Characteristics at Baseline (Patients With CTCAE Adverse Event Data Available) eFigure 1. Cumulative Incidence of Biochemical Failure (HDR-BT vs 2-Fraction SBRT vs 5-Fraction SBRT) eFigure 2. Cumulative Incidence of Distant Metastases, Cause-Specific Survival, and Overall Survival eFigure 3. EPIC Domain Scores Changed From Baseline Over Time: Urinary Domain, Bowel Domain, Sexual Domain, and Hormonal Domain

Supplement 2.
Data Sharing Statement
