# Error in Figure Labels

**DOI:** 10.1001/jamanetworkopen.2026.5720

**Published:** 2026-03-20

In the Original Investigation titled “SBRT vs HDR Brachytherapy for Intermediate-Risk Prostate Cancer,”^1^ published February 25, 2026, “adverse events” in panel titles and y-axis labels in Figure 2 was mislabeled as “toxic effects.” The article has been corrected.^1^
