# Supplementary material for: SBRT vs HDR Brachytherapy for Intermediate-Risk Prostate Cancer
Source: JAMA Netw Open. 2026 Feb 25;9(2):e260146. doi: 10.1001/jamanetworkopen.2026.0146 (PMC12936880; doi:10.1001/jamanetworkopen.2026.0146)
Supplement: Supplement 1. — eTable 1. Cumulative Incidence of Biochemical Failure (HDR-BT vs 2-Fraction SBRT vs 5-Fraction SBRT) eTable 2. Univariate Analysis for Biochemical Failure eTable 3. Multivariable Analysis for Biochemical Failure eTable 4. Demographic and Clinical Characteristics at Baseline (Patients With CTCAE Adverse Event Data Available) eFigure 1. Cumulative Incidence of Biochemical Failure (HDR-BT vs 2-Fraction SBRT vs 5-Fraction SBRT) eFigure 2. Cumulative Incidence of Distant Metastases, Cause-Specific Survival, and Overall Survival eFigure 3. EPIC Domain Scores Changed From Baseline Over Time: Urinary Domain, Bowel Domain, Sexual Domain, and Hormonal Domain [file jamanetwopen-e260146-s001.pdf]

Supplemental Online Content

Udovicich C, Cheung P, Chu W, et al. SBRT vs HDR brachytherapy for intermediate-risk prostate cancer. *JAMA Netw Open*. 2026;9(2):e260146.
doi:10.1001/jamanetworkopen.2026.0146

eTable 1. Cumulative Incidence of Biochemical Failure (HDR-BT vs 2-Fraction SBRT vs 5-Fraction SBRT)
eTable 2. Univariate Analysis for Biochemical Failure
eTable 3. Multivariable Analysis for Biochemical Failure
eTable 4. Demographic and Clinical Characteristics at Baseline (Patients With CTCAE Adverse Event Data Available)
eFigure 1. Cumulative Incidence of Biochemical Failure (HDR-BT vs 2-Fraction SBRT vs 5-Fraction SBRT)
eFigure 2. Cumulative Incidence of Distant Metastases, Cause-Specific Survival, and Overall Survival
eFigure 3. EPIC Domain Scores Changed From Baseline Over Time: Urinary Domain, Bowel Domain, Sexual Domain, and Hormonal Domain

This supplemental material has been provided by the authors to give readers additional information about their work.

© 2026 Udovicich C et al. *JAMA Network Open*.

eTable 1: Cumulative Incidence of Biochemical Failure (HDR-BT vs. two-fraction SBRT vs. five-fraction SBRT)

	HDR-BT (n = 67)	Two-fraction SBRT (n = 49)	Five-fraction SBRT (n = 131)	p-value
**Cumulative incidence of biochemical failure**				**0.0007**
At 5-year (95% CI)	7.8% (1.0-14.6%)	4.1% (0-9.8%)	2.5% (0-5.3%)	
At 8-year (95% CI)	24.0% (11.4-36.6%)	7.4% (0-15.9%)	6.2% (1.2-11.3%)	
At 10-year (95% CI)	38.0% (19.8-56.1%)	7.4% (0-15.9%)	10.5% (3.5-17.6%)	
Two-fraction SBRT vs. five-fraction SBRT	p=0.87			
HDR vs. two-fraction SBRT	p=0.03			
HDR vs. five-fraction SBRT	p<0.01			

© 2026 Udovicich C et al. *JAMA Network Open*.

eTable 2: Univariate Analysis for Biochemical Failure

	**Univariate**	
	**HR (95% CI)**	**p-value**
**Treatment**<br>SBRT<br>HDR-BT	 1<br>3.69 (1.80-7.55)	**0.0004**
**Treatment categories**		**0.0017**
Five-fraction SBRT<br>HDR-BT	1<br>3.73 (1.70-8.16)	**0.001**
Five-fraction SBRT<br>Two-fraction SBRT	1<br>1.05 (0.28-3.87)	0.94
Two-fraction SBRT<br>HDR-BT	1<br>3.55 (1.05-12.06)	**0.04**
**Age (years)**	1.02 (0.97-1.07)	0.48
**T Stage**<br>1a-c<br>2a-c	 1<br>1.42 (0.68-2.97)	0.35
**PSA at baseline**<br><10<br>≥10	 1<br>2.33 (1.16-4.68)	**0.02**
**ISUP grade group**		0.39
3+3<br>3+4	1<br>0.87 (0.23-3.32)	0.84
3+3<br>4+3	1<br>1.58 (0.36-6.85)	0.54
3+4<br>4+3	1<br>1.81 (0.78-4.21)	0.17
**Cores positive**<br><50%<br>≥50%	 1<br>0.27 (0.08-0.88)	**0.03**
**NCCN intermediate-risk group**<br>Favorable<br>Unfavourable	 1<br>1.21 (0.60-2.44)	0.59

© 2026 Udovicich C et al. *JAMA Network Open*.

eTable 3. Multivariable Analysis for Biochemical Failure

**Variable**	**HR (95% CI)**	***P* value**
Treatment		
SBRT	1 [Reference]	<.001
HDR-BT	5.26 (2.46-11.25)	
T stage		
1a-c	1 [Reference]	.07
2a-c	1.98 (0.93-4.23)	
PSA at baseline, ng/mL		
<10	1 [Reference]	<.001
≥10	3.86 (1.81-8.22)	
ISUP grade group		
3 + 3	1 [Reference]	.16
3 + 4	4.28 (0.55-33.3)	
3 + 3	1 [Reference]	.07
4 + 3	6.98 (0.82-59.29)	
3 + 4	1 [Reference]	.25
4 + 3	1.63 (0.70-3.81)	

Abbreviations: HDR-BT, high–dose-rate brachytherapy; HR, hazard ratio; ISUP, International Society of Urological Pathology; PSA, prostate-specific antigen; SBRT, stereotactic body radiotherapy.

© 2026 Udovicich C et al. *JAMA Network Open*.

eTable 4: Demographic and Clinical Characteristics at Baseline (patients with CTCAE adverse event data available)

	HDR-BT (n = 67)	SBRT (n = 60)	p-value
**Age (years)**			
Mean (range)	66.0 (49-80)	67.2 (56-80)	0.38
**Clinical stage**			
T1a-c	52 (77.6%)	42 (70.0%)	0.06
T2a	15 (22.4%)	12 (20.0%)	
T2b	0	4 (6.7%)	
T2c	0	2 (3.3%)	
**PSA at baseline**			
Median (Q1, Q3)	6.4 (4.8, 8.8)	6.9 (4.8, 8.8)	0.89
**PSA at baseline**			
<10	55 (82.1%)	53 (88.3%)	0.32
≥10	12 (17.9%)	7 (11.7%)	
**ISUP Grade Group (GG)**			
GG1: GS 3+3	3 (4.5%)	2 (2.3%)	0.93
GG2: GS 3+4	57 (85.1%)	50 (83.3%)	
GG3: GS 4+3	7 (10.5%)	8 (13.3%)	
**NCCN Risk group**			
Favorable Intermediate risk	41 (61.2%)	30 (50.0%)	0.20
Unfavorable Intermediate risk	26 (38.8%)	30 (50.0%)	
**Prostate volume at baseline, cc**			
Median (Q1, Q3)	35 (27, 43)	37 (30, 44)	0.32
**IPSS Score at baseline**			
Median (Q1, Q3)	4 (2, 8)	5 (3, 8)	0.36

© 2026 Udovicich C et al. *JAMA Network Open*.

eFigure 1: Cumulative Incidence of Biochemical Failure (HDR-BT vs. two-fraction SBRT vs. five-fraction SBRT)

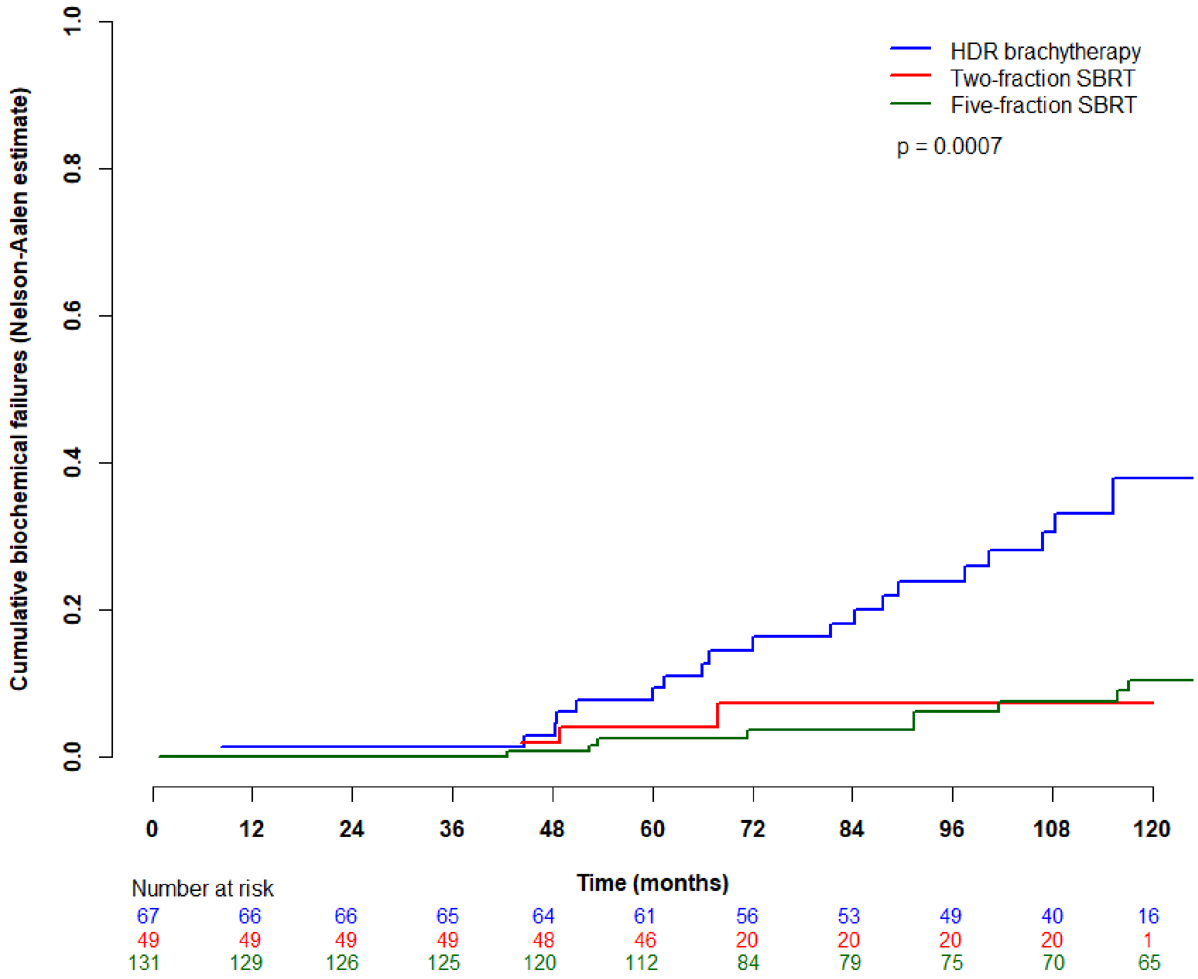

© 2026 Udovicich C et al. *JAMA Network Open*.

eFigure 2

A: Cumulative Incidence of Distant Metastases

B: Cause-specific Survival

C: Overall Survival

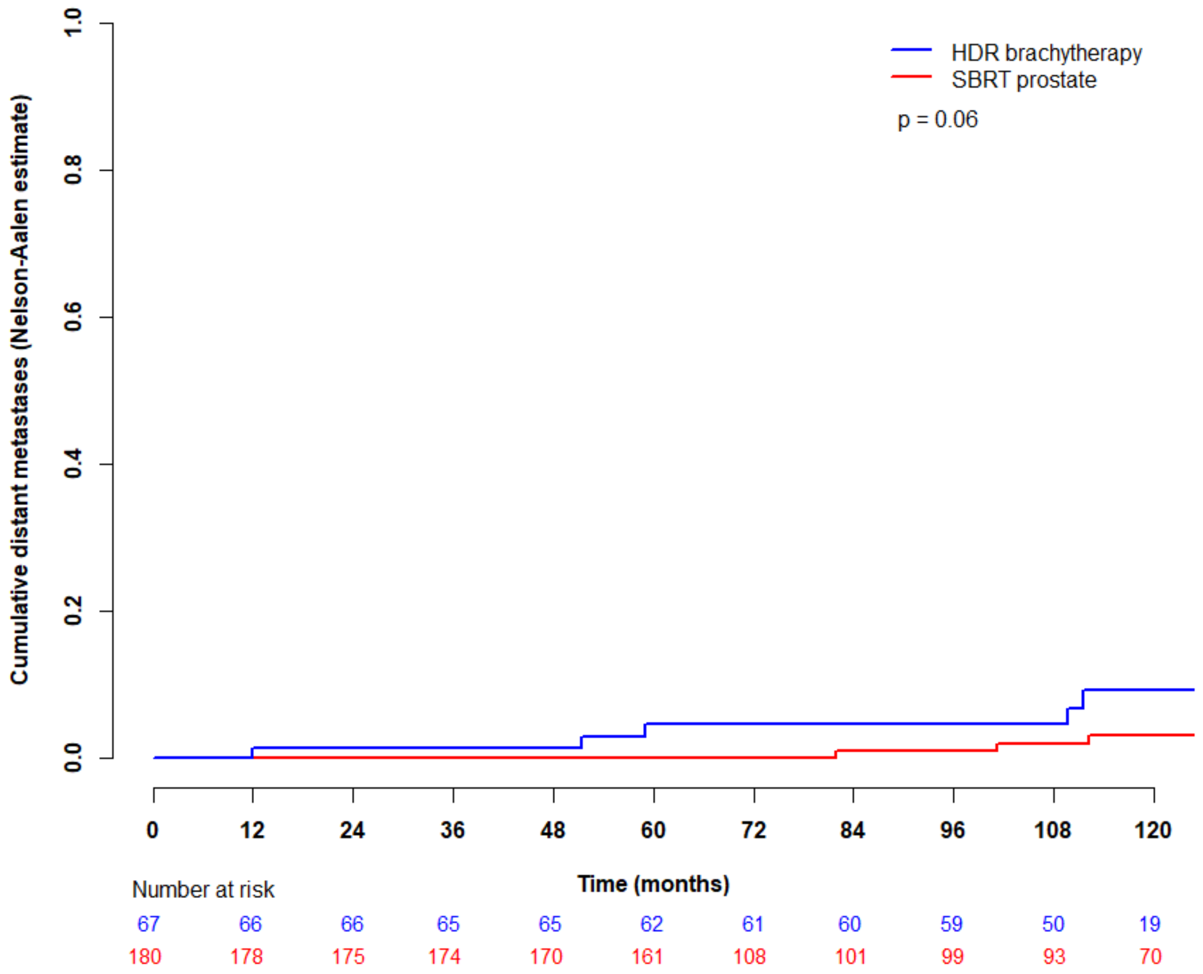

© 2026 Udovicich C et al. *JAMA Network Open*.

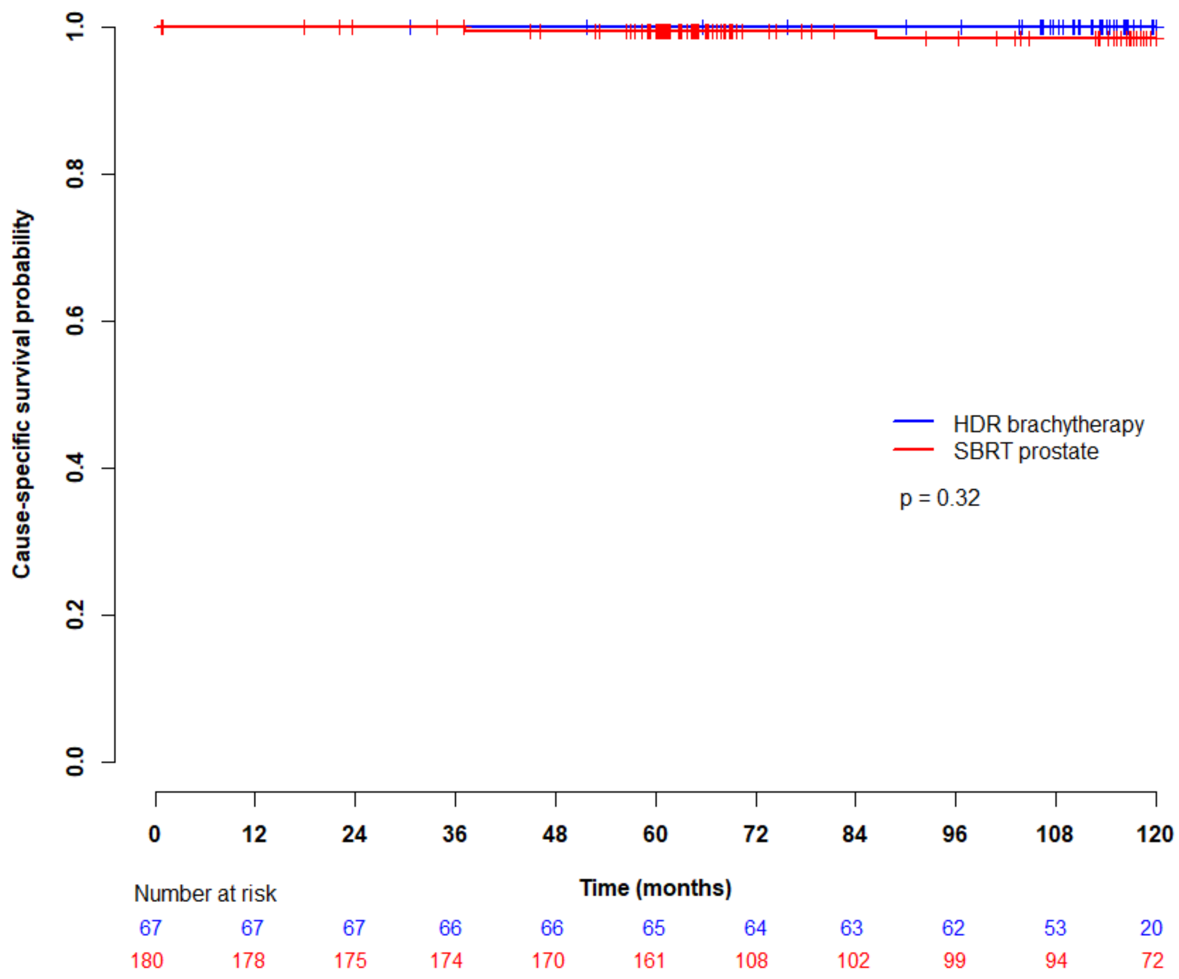

© 2026 Udovicich C et al. *JAMA Network Open*.

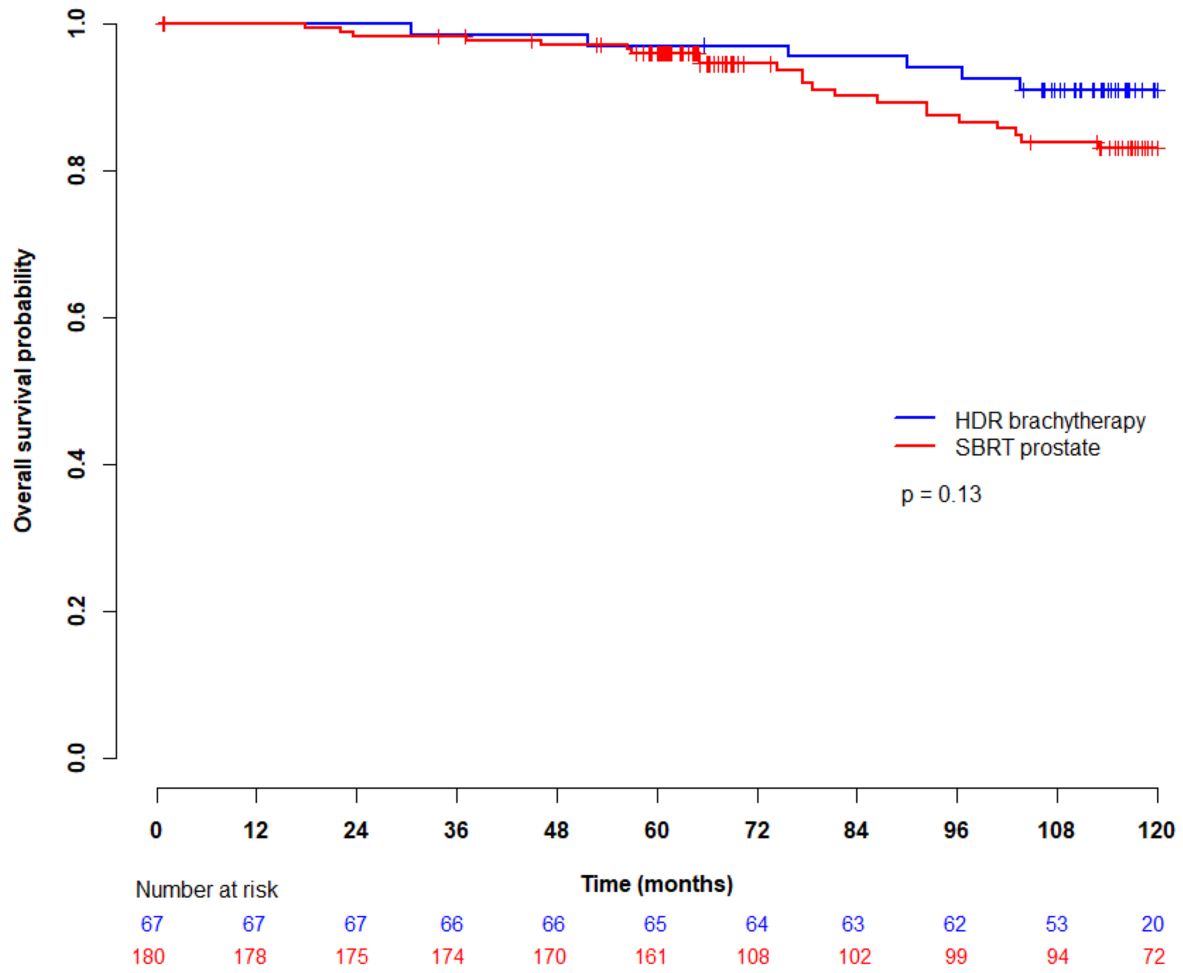

© 2026 Udovicich C et al. *JAMA Network Open*.

eFigure 3: EPIC Domain Scores Changed from Baseline Over Time
A: Urinary Domain
B: Bowel Domain
C: Sexual Domain
D: Hormonal Domain

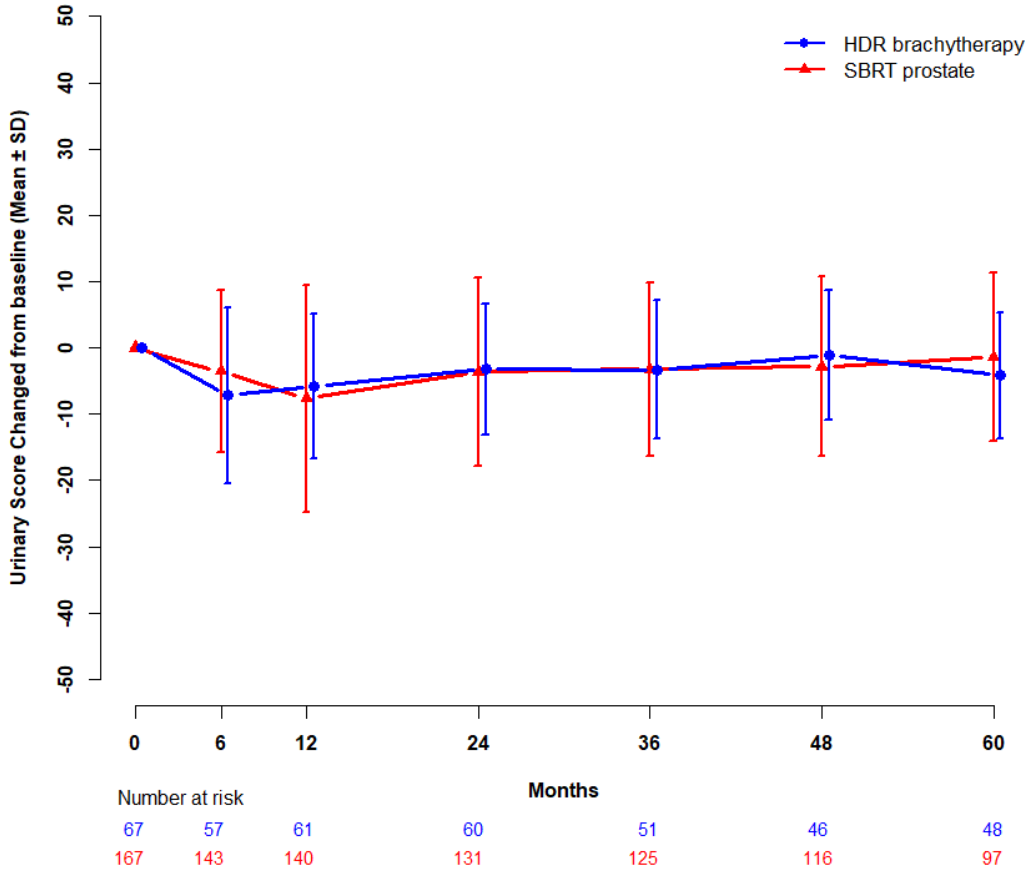

© 2026 Udovicich C et al. *JAMA Network Open*.

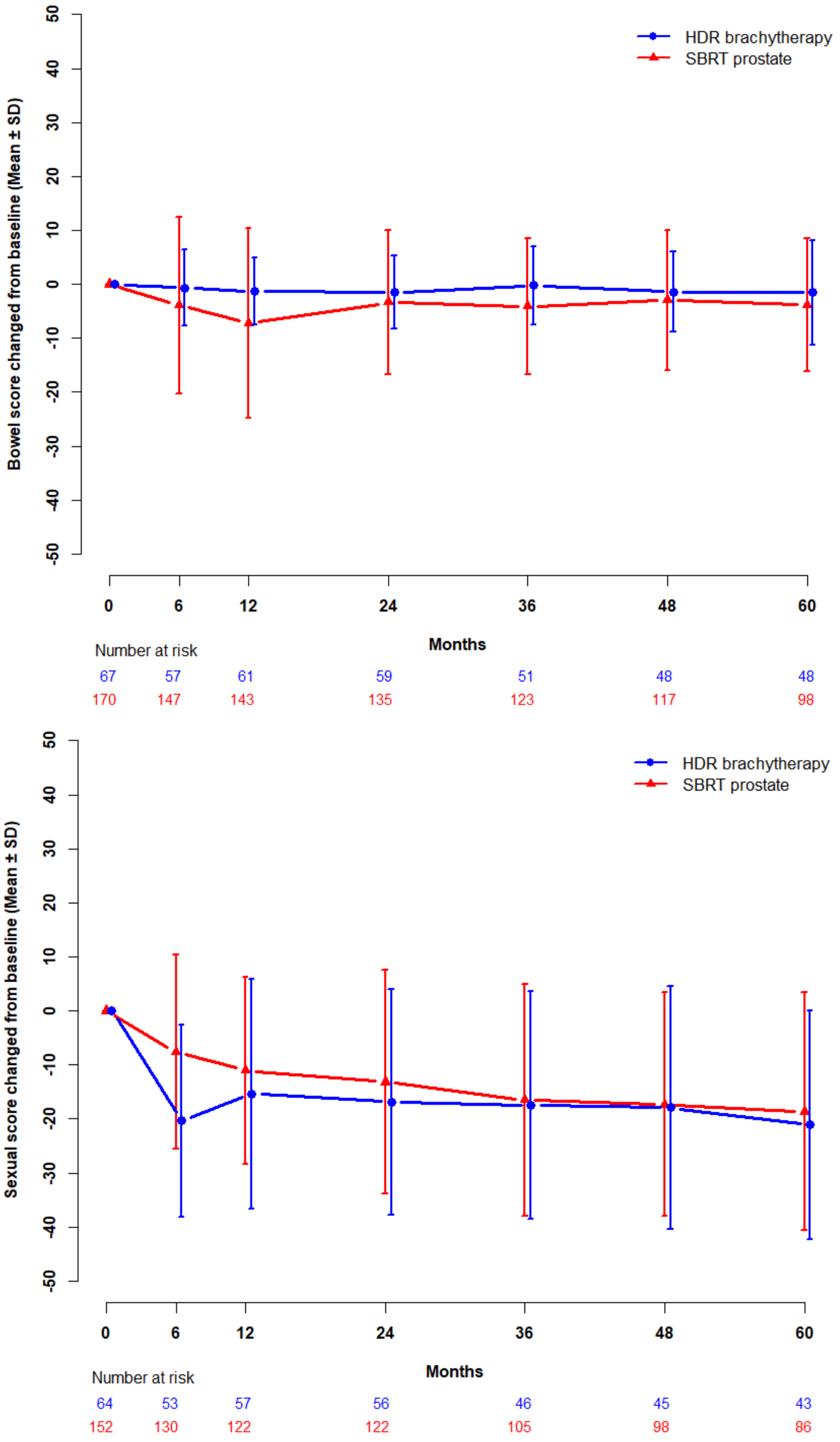

© 2026 Udovicich C et al. *JAMA Network Open*.

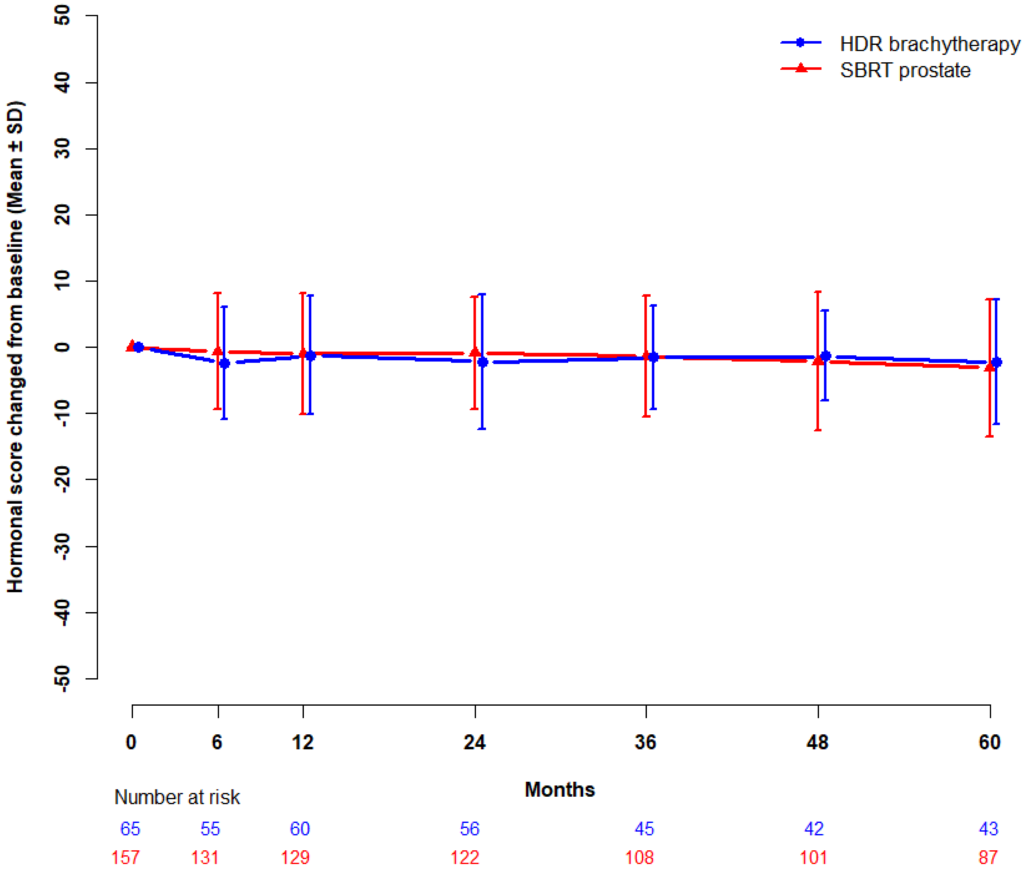

© 2026 Udovicich C et al. *JAMA Network Open*.
