# Supplementary material for: SBRT vs HDR Brachytherapy for Intermediate-Risk Prostate Cancer
Source: JAMA Netw Open. 2026 Feb 25;9(2):e260146. doi: 10.1001/jamanetworkopen.2026.0146 (PMC12936880; doi:10.1001/jamanetworkopen.2026.0146)
Supplement: Supplement 2. — Data Sharing Statement [file jamanetwopen-e260146-s002.pdf]

Data Sharing Statement

Udovicich. SBRT vs HDR Brachytherapy for Intermediate-Risk Prostate Cancer. *JAMA Netw Open*. Published online February 25, 2026. doi:10.1001/jamanetworkopen.2026.0146

Data
Data available: No
